# Alterations in Cerebrospinal Fluid Proteins in a Presymptomatic Primary Glioma Model

**DOI:** 10.1371/journal.pone.0049724

**Published:** 2012-11-19

**Authors:** John C. Whitin, Taichang Jang, Milton Merchant, Tom T-S. Yu, Kenneth Lau, Benjamin Recht, Harvey J. Cohen, Lawrence Recht

**Affiliations:** 1 Department of Pediatrics, Stanford University School of Medicine, Stanford, California, United States of America; 2 Department of Neurology, Stanford University School of Medicine, Stanford, California, United States of America; 3 The Canary Center, Department of Radiology, Stanford University School of Medicine, Stanford, California, United States of America; 4 Department of Computer Sciences, University of Wisconsin, Madison, Wisconsin, United States of America; Ospedale Pediatrico Bambino Gesu’, Italy

## Abstract

**Background:**

Understanding the early relationship between brain tumor cells and their environment could lead to more sensitive biomarkers and new therapeutic strategies. We have been using a rodent model of neurocarcinogenesis in which all animals develop brain tumors by six months of age to establish two early landmarks in glioma development: the appearance of a nestin^+^ cell at thirty days of age and the appearance of cellular hyperplasia between 60 and 120 days of age. We now report an assessment of the CSF proteome to determine the changes in protein composition that occur during this period.

**Materials and Methods:**

Nestin^+^ cell clusters and microtumors were assessed in 63 ethylnitrosourea-exposed rats on 30, 60, and 90 days of age. CSF was obtained from the cisterna magna from 101 exposed and control rats at 30, 60, and 90 days and then analyzed using mass spectrometry. Differentially expressed peaks were isolated and identified.

**Results:**

Nestin^+^ cells were noted in all ethylnitrosourea-exposed rats assessed pathologically. Small microtumors were noted in 0%, 18%, and 67% of 30-, 60-, and 90-day old rats, respectively (*p*<0.05, Chi square). False Discovery Rate analysis of peak intensities showed that the number of true discoveries with p<0.05 increased markedly with increasing age. Isolation and identification of highly differentially detected proteins at 90 days of age revealed increases in albumin and a fragment of α1 macroglobulin and alterations in glutathionylated transthyretin.

**Conclusions:**

The presence of increased albumin, fragments of cerebrospinal fluid proteins, and glutathione breakdown in temporal association with the development of cellular hyperplasia, suggests that, similar to many other systemic cancers, inflammation and oxidative stress is playing an important early role in the host’s response to brain tumor development and may be involved in affecting the early growth of brain tumor.

## Introduction

The influence of the local environment in the development of cancer has been clearly established for several systemic neoplasms including colon, breast and prostate cancers [1]–[3]. In contrast, study of these early relationships in developing brain cancers has been significantly limited by the relative inaccessibility of this tissue.

We believe that a better understanding of the ongoing *in situ* environmental changes preceding the development of clinical abnormalities may lead to novel diagnostic and therapeutic strategies in primary brain tumors. Although it would be difficult to visualize tumors at very early stages in brain parenchyma, cerebrospinal fluid (CSF) represents a readily accessible source that could serve as a reporter of early stages of tumor development. Approximately 10–30% of all CSF is extrachoroidal in origin and is represented by bulk flow of the interstitial fluid from brain parenchyma into the ventricles and subarachnoid space [4], . To date, however, studies have almost exclusively examined samples drawn from patients in whom the brain tumor is already clinically evident, which makes it difficult to distinguish what is a result of the brain tumor itself versus other effects including the impact of a space occupying lesion and blood brain barrier disruption.

Surface-enhanced laser desorption/ionization TOF mass spectrometry (SELDI TOF MS) has been used successfully to identify biomarkers in blood from various malignancies using comparative proteomic strategies [6]–[9]. Nevertheless, while there have been several clinical studies that have attempted to identify biomarkers of brain tumor using comparative proteomic techniques, they all suffer from an inability to control such factors as age, space occupying volume and tissue permeability, thus obscuring whether a changed protein expression pattern accurately represents an effect of the neoplastic process.

In order to control for these variables, we assessed changes in CSF protein composition during the period in which brain tumors develop after a single *in utero* exposure to the neurocarcinogen ethylnitrosourea (ENU). Numerous pathological studies including those from our laboratory have established that gliomas invariably develop in this model. While the gliomas are not generally detectable pathologically until approximately 90 days of age (P90), and even later using available magnetic resonance imaging (MRI) technology, clear landmarks of developing tumors can be noted as early as P30 [10]–[12]. By obtaining adequate amounts of CSF via intracisternal puncture, we assessed changes in the CSF proteome at days P30, P60 and P90 using SELDI/TOF MS.

In this controlled paradigm in which matched ENU- and saline-exposed rats were examined, we demonstrate proteomic changes in CSF as early as P60, which increase by P90 in ENU-exposed rats. Furthermore, the identification of changes in glutathionylated products of transthyretin as well as a fragment of α1-macroglublin as two of the most significant changes that correlated with the development of early cellular hyperplasia suggests that increased proteolysis is present within the brain environment during a time before tumors are detectable by imaging.

## Results

### Development of Brain Tumors in Progeny of ENU-exposed rats

**Figure 1:**
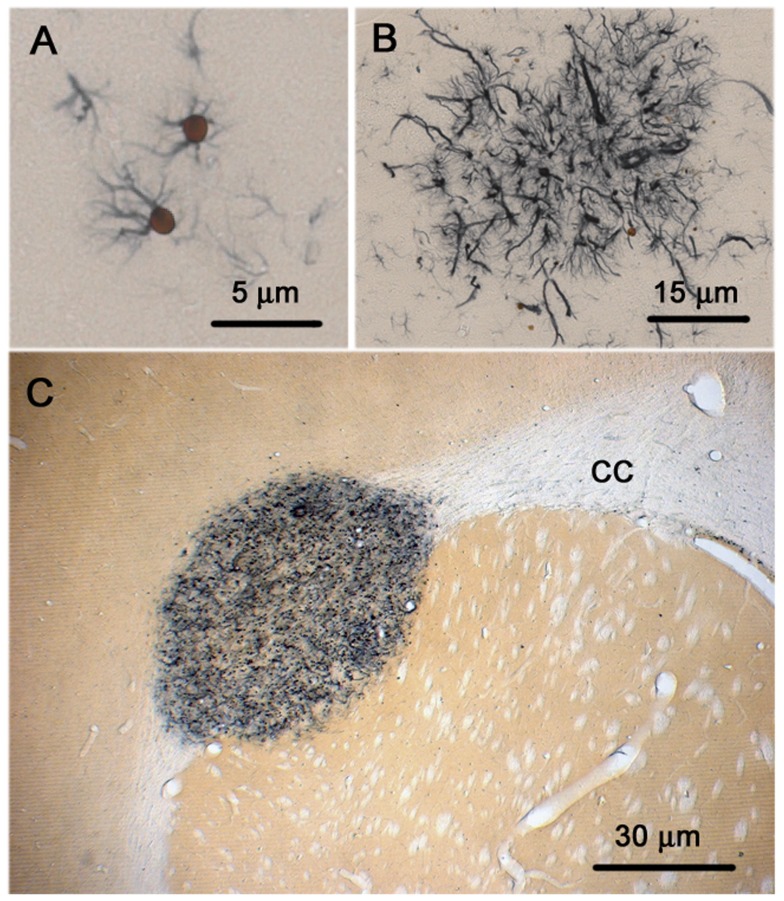
Nestin^+^ cells in glioma development after *in utero* ENU exposure. (A) Characteristic appearance of nestin+ cells that arise in locations commonly noted to harbor tumors at later stages. These usually appear as either a single or more nestin+ cells that arise on a bland background that appears normal on H&E staining. These clusters sometimes can include dozens of cells (B). (C) Small microtumor arising in the external capsule of the *corpus callosum* (CC). These tumors are hypercellular and apparent on H & E and contain a significant fraction of cells that do not express neural markers.

**Figure 2:**
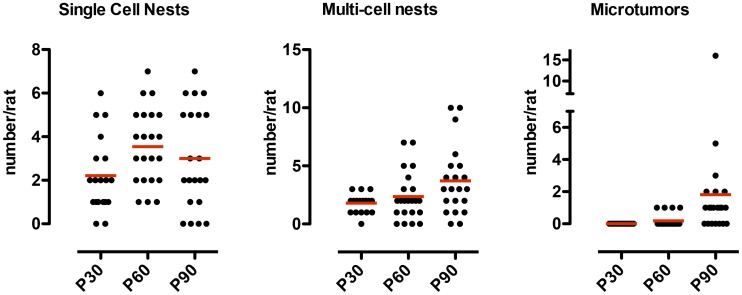
Numbers of nests and hyperplastic microtumors as a function of age. The number of tumor nodules is significantly greater at P90 (67%) than P60 (18%) and P30 (0%) (*p*<0.001, Chi-square).

ENU exposed rats (n = 63) (20 from P30, 22 from P60 and 21 from P90) were examined histologically for the presence of nestin^+^ precursor lesions (nests) as well as microtumors (areas of cellular hyperplasia measuring less than 200 µm) as previously described [10]. Consistent with previous reports [10]–[12], precursor nests were noted in all rats at all three ages (100%) (**Figure 1**). In contrast, microtumors were not noted in any rats sacrificed at P30, only 4 rats (18%) at P60 and 67% of rats at P90 (*p*<0.001, Chi-square, compared to other two groups) (**Figure 2**). No macroscopic tumors were found in any animals at the time points examined. Previously we had demonstrated that these nestin+ cells and microtumors are negative for neuronal markers such as βIII-tubulin [10].

### Differential Protein Expression in CSF Identified by SELDI TOF MS

CSF was collected from a total of 51 ENU and 50 saline exposed rats over three independent experiments. At P30 (13 ENU- and 11 saline-treated), P60 (16 ENU- and 16 saline-treated) and P90 (22 ENU- and 23 saline-treated), mass spectra of CSF applied to CM10 ProteinChip arrays were collected for the three postpartum ages (P30, P60, and P90) as described in **Methods**. The relative intensities of peaks were different in the CSF of rats obtained at these three ages. For this reason we grouped the spectra by postpartum age for baseline correction, noise reduction and intensity normalization. The spectra for all three ages were then grouped together for the purpose of finding peaks, and then separated again by age for further analysis of the peaks at each age.

**Figure 3:**
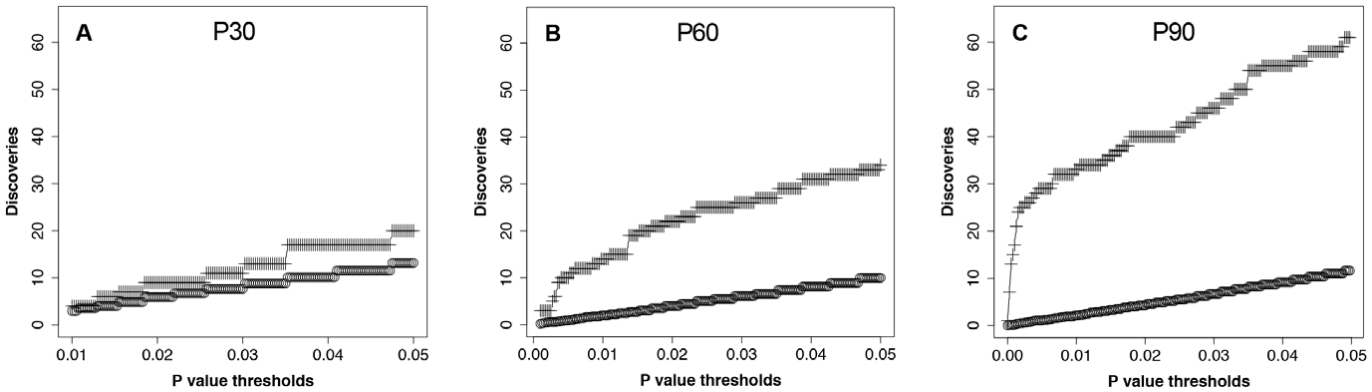
The number of total discoveries relative to global false discoveries as a function of increasing age. The top line (+) indicates the number of discoveries observed at each *p*-value up to 0.05. The bottom line (o) represents the average number of discoveries observed at each *p* value when the data are permuted 100 times.

We identified 247 peaks and determined the number of peaks that differed significantly in ENU-exposed vs. control rats at each age (i.e., P30, P60 and P90). We noted that the number of peaks that were significantly different (i.e., *p*<0.05, Mann Whitney *U*-test comparison) increased from 20 to 34 to 61 for P30, P60 and P90, respectively (**Table 1**). When examining large numbers of peaks, however, it is important to address the problem of discovering false positives. When the number of peaks discovered with *p*<0.05 is therefore compared to the number obtained when the results are permuted to determine false discoveries, the differences between the number of peaks discovered and the average global false discoveries increased with age (**Figure 3**).

We further assessed our results by determining the local false discovery rate (FDR) for each peak. Using this approach, we observed that despite there being 21 (of 247) peaks that appeared to be significantly different at P30, the lowest local FDR of 0.28 for any peak indicated that all these peaks with *p*<0.05 might have been found due to chance. The number of significantly different peaks increases at P60 and P90. At the latter P90 age, 25 peaks had a local FDR of less than 0.05 with Mann-Whitney *U* test values *p*<0.002 (**Figure 3** and **Table 1**). This FDR analysis supports the contention that most of the peaks noted at P90 with very low *p*-values were true discoveries.

**Table 1:** Mann-Whitney *U* test results with Local FDR analysis.

	Number of peaks with specified local FDR at each age		
	P30	P60	P90
Local FDR<0.25	0	19	54
Local FDR<0.10	0	10	26
Local FDR<0.05	0	1	25

247 peaks analyzed.

### Identification of Differentially Express Proteins

**Figure 4:**
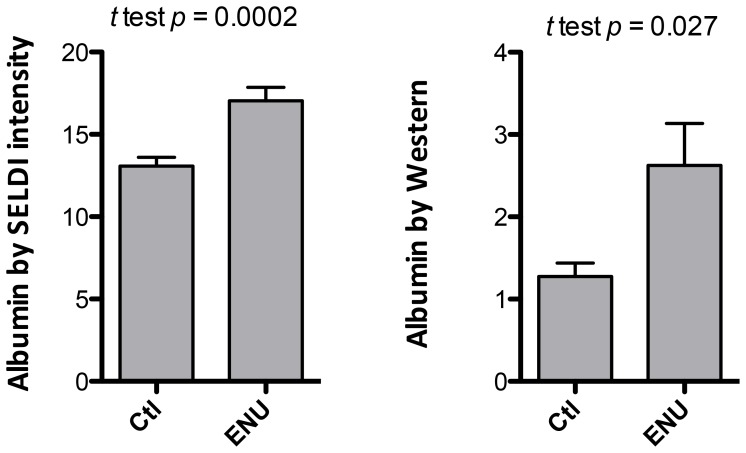
Albumin levels in P90 ENU-exposed rat CSF by SELDI peak intensity and Western blotting. Left panel represents the SELDI peak intensities (µA signal) (n = 23 for Ctl, n = 22 for ENU, mean±SEM, Mann-Whitney *U* test). Right panel represents results of Western blots, measured using densitometry (units related to the integration of absorbance by the band on x-ray film, with the integration area fixed to be the same size for all lanes) (n = 7 for Ctl, n = 7 for ENU, mean±SEM, Mann-Whitney *U* test).

The first 27 P90 peaks with MW U-test of *p*<0.05 and a local FDR<0.12 are listed in **Table 2**. Using clustering software to identify aliases in the data (e.g. the same peak observed with different laser energies, or the same proteins with different charge), we focused on four proteins of sufficient intensity to enable purification. The peak intensities for albumin were significantly elevated in ENU-exposed rats and are represented in the table as peaks of the same protein but with different charges (z = 1∶66,100, z = 2∶33,109, z = 3, 22,034) and albumin dimers (peak 131,938). The increase in the intensity of the 66,100 peak in the ENU group was confirmed by Western analysis of CSF using anti-albumin antibody (**Figure 4**).

**Table 2:** Peaks with low Mann-Whitney *U* test and low local FDR.

m/z	laser energy	note	ENU mean	ENU sem	Ctl mean	Ctl sem	*p* value	local FDR
*6909*	med	z = 2 for 13840	9.97	0.28	8.18	0.25	0.000008	0.0014
*6859*	med	z = 2 for 13725	14.07	0.61	10.61	0.43	0.000029	0.0025
*22054*	med	z = 3 for 66110	1.00	0.09	0.55	0.05	0.000038	0.0025
**66110**	high	**Albumin**	17.04	0.82	13.08	0.52	0.000084	0.0054
46643	high		0.24	0.04	0.46	0.04	0.000105	0.0054
**13725**	med	**transthyretin (Cys-)**	74.09	2.76	58.45	2.31	0.000131	0.0054
**3493**	low	**α1-macroglobulin fragment**	12.17	2.28	6.51	1.66	0.000201	0.0078
*27410*	med	dimer of 13725	0.59	0.03	0.45	0.03	0.000273	0.0078
**22893**	high	**PGD2S**	17.94	1.09	22.97	0.84	0.000334	0.0078
21365	high		3.39	0.17	4.20	0.13	0.000369	0.0078
*6823*	med	z = 2 for 13670	9.85	0.40	7.61	0.37	0.000407	0.0078
*33109*	high	z = 2 for 66110	5.24	0.22	4.14	0.16	0.000407	0.0078
36781	high		0.74	0.07	1.10	0.06	0.000449	0.0078
14120	med		24.90	1.19	31.01	1.57	0.000658	0.0161
110080	high		0.03	0.00	0.04	0.00	0.000722	0.0161
**13670**	med	**? transthyretin (sulfonated)**	49.70	1.99	38.96	2.08	0.000792	0.0161
*131938*	high	dimer of 66110	0.76	0.09	0.44	0.04	0.000868	0.0161
3504	low		2.75	0.31	1.80	0.29	0.001041	0.0161
38453	high		0.65	0.04	0.83	0.03	0.001041	0.0161
21593	high		3.30	0.17	4.04	0.12	0.001138	0.0161
*6795*	med	z = 2 for 13601	16.26	0.47	13.89	0.45	0.001244	0.0161
**13913**	med	**transthyretin (glutathionylated)**	122.75	5.28	143.62	6.35	0.001358	0.0161
27538	med		0.81	0.03	0.68	0.03	0.001482	0.0161
2887	low		2.38	0.20	1.47	0.13	0.001482	0.0161
*6865*	low	z = 2 for 13725	11.02	0.73	8.53	0.58	0.001760	0.0478
**13788**	med	**transthyretin (-Cys-Gly)**	51.29	1.34	44.48	1.64	0.002265	0.0868
**13601**	med	**transthyretin (unmodified)**	80.66	2.00	70.86	2.33	0.003398	0.1167

Peaks in bold type are z = 1. Peaks in italics are aliases (z = 2 or 3, or dimers) of the indicated protein.

**Figure 5:**
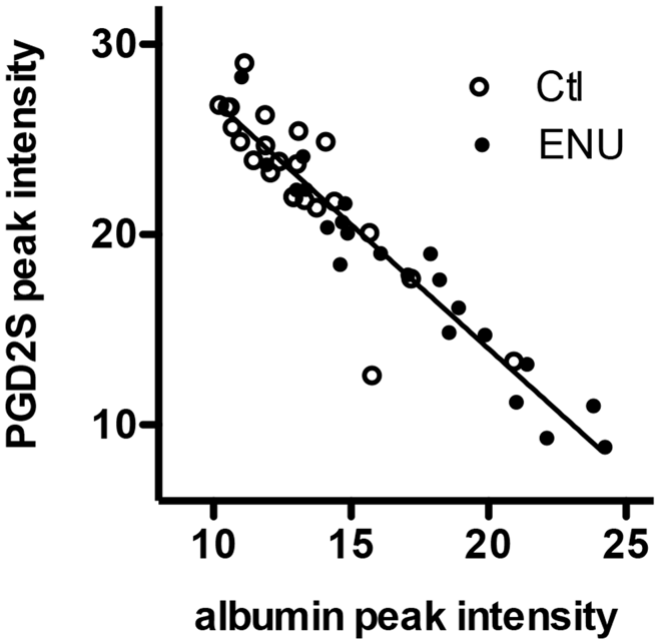
Relationship between albumin and PGD2S peak intensities. This relationship was noted for both ENU-exposed and control rats at all ages. The linear regression correlation coefficient was R = −0.945.

Another protein of interest had peak intensities at m/z 22,893 that were significantly lower in the CSF of ENU-exposed than control rats. This peak was then identified by purification and sequence analysis of tryptic peptides. Based on the identification of the peak as prostaglandin D2 Synthase (PGD2S) (**Figure S2**), we performed Western analysis and slot blot immunoassay using anti-PGD2S to verify decreased protein expression. These techniques did not verify the decreased amount of PGD2S in ENU relative to control CSF that were noted using peak intensities. We hypothesize that the increased albumin present in the CSF in ENU rats suppressed the ionization or detection of PGD2S by mass spectrometry, resulting in an artifactual decrease in the PGD2S peak intensity. There was a near-perfect inverse correlation between the albumin peak intensity and the 22,893 peak intensity (**Figure 5**).

**Figure 6:**
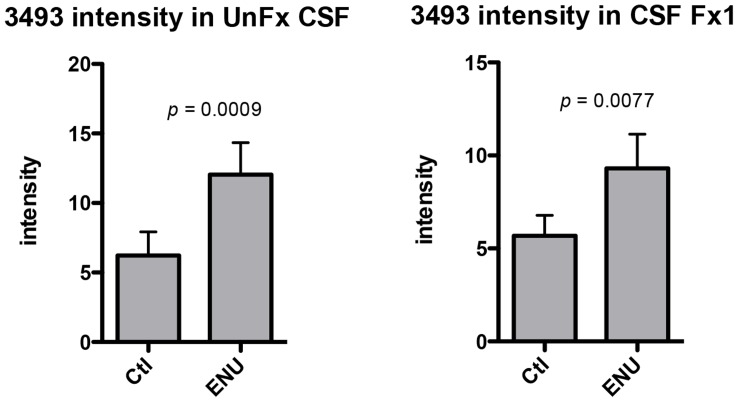
SELDI peak intensities of 3493. The SELDI peak intensities for the 3493 peak in P90 CSF are shown for both A) unfractionated CSF (n = 23 for Ctl, n = 22 for ENU, mean±SEM, Mann Whitney *U* test); and B) F×1 of anion exchange-fractionated CSF (n = 20 for Ctl, n = 20 for ENU, mean±SEM, Mann-Whitney *U* test).

A third peak (m/z 3493) was also higher in the ENU rats than in the control rats. To decrease the complexity of the CSF, we also fractionated the CSF samples by strong anion-exchange chromatography prior to acquisition of SELDI spectra (see **Methods)**. The m/z 3493 peak was found in the pass-through fraction (F×1) and therefore did not bind to the anion exchange media at pH 9, suggesting the peptide had a high pI. The peak intensities of the m/z 3493 peak in the pH 9 fraction were still higher in the ENU than the control rats **(** **Figure 6** **)**. The pH 9 fraction did not contain any albumin, thus confirming its peak intensities were not affected by the presence of albumin. The m/z 3493 peptide was purified and identified as a novel fragment of α1-macroglobulin by both direct sequence analysis (MALDI MS/MS), and by sequence analysis of tryptic peptides (**Figure S3**). The sequence of the 3493 peptide is SFSYKPRAPSAEVEMTAYVLLAYLTSASSRPT, which corresponds to amino acids 1212–1243 of rat α-1-macroglobulin (a 1500 amino acid protein). Of note, a 45 kDa subunit of α-1-macroglobulin has been reported, from amino acids 1245–1500. Amino acid 1244 is the amino acid arginine. Neither in the discovery spectra, nor during the purification, did we find evidence of a peptide with an m/z 3650, which would be the theoretical m/z of the 3493 peptide with an additional C-terminal arginine.

Fourteen of the 61 peaks with *p*<0.05 were related to each other as either aliases (different charges) or post-translational modifications of transthyretin. The peaks were purified, characterized and identified. The peaks 13601, 13670, 13725, 13788, and 13913 (and their aliases) were purified as a group and extracted from a single band on non-reduced SDS-PAGE (**Figure S4**). Hypothesizing that these peaks were the unmodified and various modifications of transthyretin, we digested one part of the gel band with the enzyme Arg C without reduction and alkylation, and another part of the band with Arg C after proteins in the band had been reduced and alkylated. The 13601 peak corresponds to unmodified transthyretin, while the identities of the other peaks were different sulfhydryl modifications of the lone cysteine within the primary structure of rat transthyretin. The 13725 peak was found to be cysteinylated transthyretin; the 13788 peak, the –Cys-Gly modified transthyretin; and the 13913 peak, the glutathionylated transthyretin. Peptides consistent for the unmodified parent transthyretin and these post-translational sulfhydryl modifications were found in the mass spectrum of the Arg C-digested non-reduced band. Peptides for only the parent, unmodified transthyretin were found in the reduced/alkylated Arg C-digested band, a result confirmed by sequencing of the peptide (details in Figure S4). The 13670 peak was not identified, but may correspond to sulfonated transthyretin [13].

**Figure 7:**
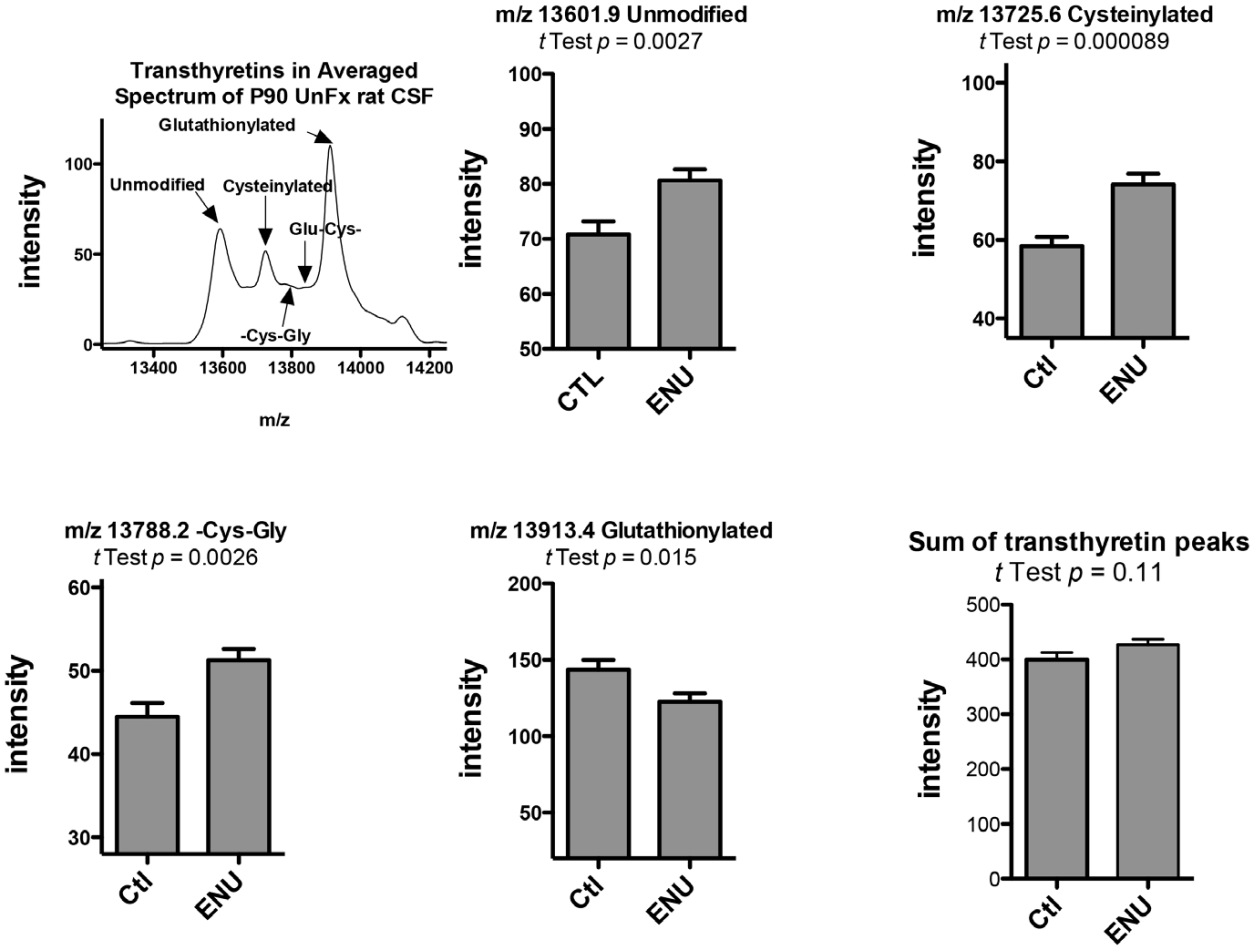
Changes in the amounts of transthyretin fragments in ENU-exposed rats compared with control rats at P90. (A) Spectrum of transthyretin peaks. (B–E). Peak intensities of unmodified, cysteinylated, -cys-gly and glutathionylated transthyretin, respectively. (F) Sum of the peak intensities of unmodified, cyteinylated, -cys-gly and glutathionylated transthyretins. (n = 23 for Ctl, n = 22 for ENU for B–F, mean±SEM, Mann-Whitney *U* test).

**Figure 7** demonstrates an averaged mass spectrum for the m/z range of 13000–14600, with the annotated identifications of the peaks. While all of these peaks were significantly different using the Mann-Whitney *U*-test (with low local FDR), there was one difference among their intensities in ENU CSF compared to the CSF of age-matched control rats. For three of the four peaks in the figure (13601, 13725, and 13788) the peak intensities in the ENU rats were higher than control rats. In contrast, the intensity of the 13913 peak (glutathionylated transthyretin) was lower in the ENU rats compared to the controls. **Figure 7** also demonstrates that the sum of intensities of the five transthyretin peaks is not significantly different when comparing the ENU and control groups. This observation is consistent with there being a change in the distribution of the various modified forms of transthyretin, but not the total amount of transthyretin.

### Formulation of a Biomarker Profile Distinguishing Early Tumor Environment

To assess the relative values of each of these proteins as potential biomarkers, we used a standard scalable algorithm, LASSO, that automatically balances classification performance against the number of markers in the classifier [14] to identify the best set of markers for classification. Our software implementation of the LASSO, based on ideas from Least-Angle-Regression [15] and Homotopy Selection [16]. The code adds one marker to the classifier at a time allowing for the manual evaluation of the tradeoff between accuracy and number of markers. Since one marker is added at a time, we can terminate the software after a classifier is fit with a desired number of markers.

**Figure 8:**
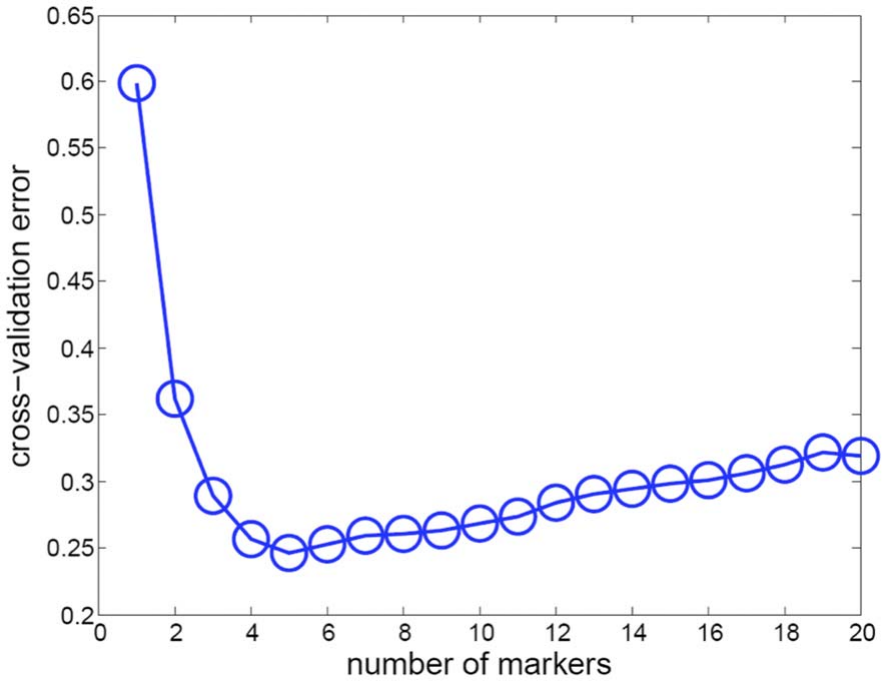
Cross-validation error versus number of biomarkers in LASSO analysis. This figure plots the average cross-validation error as the number of markers are varied. Each data point was created by running the LASSO on 80% of the data set and recording the prediction error on the held out 20%. This procedure was repeated 1000 times, and the average error is then plotted on the graph. The lowest error is achieved using a classifier with 5 markers.

Using this approach, we repeated the statistical analysis 1000 times. We subsampled 36 rats from the control/ENU population of P90 rats and computed a sequence of classifiers using the Homotopy algorithm. We observed that on the 9 held out subjects, the error was minimized using five makers (**Figure 8**). We recorded the peaks that were selected for the classifier in each round of cross-validation. **Table 3** lists the peaks that were most frequently selected in the five-marker classifiers and the frequency that they were selected in our 1000 trials. Transthyretin peaks were almost universally selected in these five-marker classifiers with only albumin also being identified in more than 50% of the analyses. No other peaks arose more than 20% of the time. Based on this analysis, therefore, we conclude that a transthyretin peak is almost always selected by the LASSO analysis, suggesting that it represents the best biomarker for distinguishing between the control and ENU data sets.

**Table 3:** LASSO Analysis.

Peak m/z	Protein ID	% Represented in Analysis
6909	**Transthyretin (Glu-Cys-) (z = 2 for 13840)**	98.7%
22054	Albumin (z = 3 for 66100)	78.1%
6859	**Transthyretin (Cys-) (z = 2 for 13725)**	75.0%
13725	**Transthyretin (Cys-)**	46.1%
6823	**Transthyretin ? (z = 2 for 13670)**	30.2%
21365	Not identified	25.7%
2886	Not identified	21.5%

## Discussion

This is the first time that the CSF protein profile has been examined prior to either the imaging or symptomatic detection of brain tumors. It is made possible by the availability of an experimental rodent model in which brain tumors invariably develop several weeks after a prenatal exposure to a neurocarcinogen. Investigations in several laboratories, including ours, have established predictable pathological landmarks including the appearance at P30 of abnormal cell and cell clusters expressing the neuroepithelial marker nestin without hyperplasia that become associated with cellular hyperplasia by P90 [10]–[12], [17]–[25].

CSF is produced mainly by the choroid plexus, an intraventricular structure formed by the invagination of the pia mater. The choroid plexus regulates chemicals in the brain not only by selectively limiting the access of blood-borne substances to the CNS, but also by serving as a unique source of essential molecules to the cerebral compartment including VEGF, IGFII transferrin, PGD2S and transthyretin [5]. In addition, up to 30% of CSF is composed of bulk flow drainage of the interstitial liquid of the nervous tissues (which lack significant lymphoid structure) into the ventricles and subarachnoid space [4]. The CSF thus represents an optimal “reporter” of the physiological and pathological status of the CNS, since both its much lower volume compared to blood, and insulation from exposure to the systemic circulation, favors the relative over-representation of brain proteins in CSF.

A major difficulty with interpreting results of CSF protein analysis, however, is that protein composition is dependent on subject age, the site where CSF is accessed (there being a two fold difference in albumin concentration between lumbar and cisternal sampling [26], [27]), the extent of blood contamination and the effects of a space occupying lesion. Thus, although there have been a number of clinical studies that have screened CSF obtained from brain tumor patients for differences in protein composition [28]–[31], it has been difficult to adequately control for these differences. Our study is therefore unique in that it is carefully balanced for subject age, site and method of CSF acquisition, as well as controlling for presence of contaminating blood (which even in minimal quantities can impact protein composition [32] (see also **Supporting Information S1**, **Figure S1**).

We chose the ages to be studied based on the staggered appearance of nestin-expressing cell clusters (which start at P30) and the association of these cells with surrounding cellular hyperplasia (which can be detected as early as P60) [10], [11]. In this series, our pathological analysis confirmed the presence of nestin^+^ cell clusters in every rat examined at all three ages (n = 63), while hyperplasias were detected in only 18% of brains examined at P60 and then 67% at P90. In association with this, we found that the global protein fingerprint was not significantly different between ENU-exposed and control subjects at P30 based on FDR analysis, after which increasing differences were noted at P60 and P90. No rat was found to have a macroscopic tumor at any of the times studied. Based on this, we conclude that CSF protein alterations noted in this study are more reflective of the cellular hyperplasia stage of this process.

To develop further insights as to the types of proteins that were altered and whether they pointed to a particular biologic process, we next identified those peaks that were most significantly different between ENU-exposed rats and controls. Several of the peaks represented albumin, which was consistently elevated in ENU-exposed rats after P60, a finding confirmed by Western blotting. Albumin levels in CSF have been noted to be elevated in patients with glioblastoma [30] and has been attributed to either disturbance of the blood brain barrier or release from tumor. Considering the microscopic size of ENU-induced hyperplasia at this age, it seems unlikely that the increased albumin reflects either albumin release from tumor cells or the impact of a space occupying lesion. Therefore, our finding would thus support Schuhmann *et al*’s conclusions that this reflects a blood brain barrier disturbance that is present long before imaging changes are apparent.

Another peak with a mass of 22.9 kD was decreased in ENU exposed rats and identified as prostaglandin D2 synthase, also known at β-trace. PGD2S is exclusively brain derived and is one of the most abundant proteins in the CSF [33], [34], representing approximately 3% of the total protein in the CSF. It is expressed in the arachnoid membrane of the leptomeninges and choroid plexus [33], [35], in oligodendrocytes and astrocytes but not mature neurons [36]. The level of PGD2S in CSF is approximately 35 × that measured in plasma [33]. Loss of PGD2S has been postulated to represent an important event in glioma progression [37] and its levels in CSF have been reported to be decreased in medulloblastoma [38].

Our subsequent studies however indicate that changes noted in PGD2S peak intensities probably reflect an artifact caused by the effect of albumin concentration on the intensity of the PGD2S peak, as supported by the observations of almost a perfect inverse correlation between the PGD2S and albumin peak intensities (**Figure 5**) and an inability to confirm a decrease in PGD2S CSF protein levels with immuno-slot blot assays. Therefore, we conclude that PGD2S is not significantly altered early in our model of brain tumor development, at a time when no macroscopic tumors are found.

The identification of an increased concentration of a cleaved fragment of α1-macroglobulin is a unique finding that points to the advantages of using a top down MS approach, since it would have not been detected using a bottom up MS/MS approach that starts with proteolytic digestion of the sample. This macroglobulin is synthesized as a single polypeptide chain that is subsequently cleaved to form heavy and light chains. The novel fragment reported here is near the C-terminus of the heavy chain that is created during processing of the full-length protein. The m/z 3493 peptide is missing the C-terminal Arg residue of the heavy chain, and no evidence of a peak representing the 3493 peptide with the additional C-terminal Arg (a theoretical m/z 3650) was found in either unfractionated or anion exchange-fractionated CSF. This macroglobulin is a broad spectrum protease inhibitor that is constitutively active (in contrast with other rat α macroglobulins, which are more acute phase reactants), present in serum and interstitial fluids and inhibits all four types of proteinases by physical entrapment [39]. The increased amounts of this novel cleaved fragment of this protease inhibitor may indicate the presence of increased protease activity in the environment of, or in reaction to, the developing brain tumor, either arising from the tumor cells themselves, a host response or both. The significant increase of a cleaved component suggests also either increased or aberrant proteolytic activity at the cellular hyperplasia stage of ENU-brain tumor development.

Finally, many of the most significantly different peaks between ENU-exposed and control rats represented different post-translational modifications of transthyretin. Transthyretin functions in the transport of both thyroxine and retinol in plasma and CSF. The disproportionately high concentration of soluble monomeric tranthyretin (14 kD) in human ventricular CSF is related to the fact that it is synthesized in high quantities within the cells of the choroid plexus [40]. Interestingly, changes in the relative levels of transthyretin in CSF have been found by SELDI in other biomarker studies of brain tumor [6], .

In an analysis of different brain tumors, transthyretin mRNA and protein were not expressed in 23 anaplastic astrocytomas and glioblastomas, being exclusively limited to choroid plexus papillomas [41]. More recently Park et al found early stage glioblastomas in humans were negative for transthyretin staining, but that advanced grade IV glioblastoma sections were positive for transthyretin staining [42]. Transthyretin was one of several serum proteins that were elevated in pateints with suspected gliomas undergoing surgery [43]. It seems unlikely that the changes in CSF transthyretin levels in this study represent release from these small nests and microtumors at this early stage of glioma development, but confirmation of this is worthy of more study. Nevertheless, we believe that the changes in this protein also highlight the capacity of the SELDI technique to detect protein fragments and other post-translational modifications. Thus, although total transthyretin levels were not found to be different between groups, there was a significant increase in the fraction of deglutathionylated transthyretin peptides in the ENU exposed rats and a concomitant decrease in glutathionylated transthyretin.

Glutathione (glutamate-cysteine-glycine; GSH) is a tripeptide utilized in disulfide exchange reactions resulting in the formation of mixed protein-glutathione disulfides. It serves as an important cellular antioxidant in the brain, where it plays a critical role in suppressing oxidative stress and maintaining cellular redox stability. In addition, glutathione can function as a storage depot for both cysteine and glutamate, thus serving an important cytoprotective function by preventing the inherent cytotoxicity of free cysteine and glutamate-dependent neuronal excitotoxicity [44].

GSH is synthesized intracellularly and frequently bound to passenger proteins upon export. It can be hydrolyzed to its γ-glutamyl moiety and cysteinylglycine, the latter of which can be further broken down in times of cellular stress so that the released amino acids can be taken up by corresponding transporters and reused by cells [45]. The capacity to cleave this γ-glutamyl bond is a unique function of the enzyme γ-glutamyl transferase (GGT), a ubiquitous enzyme located at an exo-facial position in the cellular plasma membrane primarily in tissues having secretory or absorptive functions, including the choroid plexus [46], [47] as well as cerebral microvessels, where it plays an integral role in maintaining blood brain barrier integrity [48].

In a prior study that also examined the evolution of gliomas in this experimental paradigm, we reported the consistent appearance of a unique cell that coexpressed glial fibrillary acidic protein (GFAP) and osteopontin (OPN) at the time these lesions evolved from nestin+cell clusters to hyperplastic nodules [11]. Considering that OPN is a pleiotropic molecule that plays an important role regulating inflammatory cytokine production and cell trafficking, we suggested that an important component of early glioma development involved immune activation. The results reported here are also consistent with an early host response to a developing neoplasm. Thus, the detection of both a unique peptide cleaved from α1 macroglobulin (which in its uncleaved form inhibits proteoloysis) and increased amount of deglutathionylated transthyretin suggests increased proteolysis and peptide lysis is occurring at this early juncture, probably at the level of the choroid plexus. The mechanism by which the C-terminal Arg is removed in the novel fragment of α1 macroglobulin might involve cleavage by carboxypeptidase B. Carboxypeptidase B cleaves the C-terminal Arg of osteopontin, thereby regulating neutrophil involvement in rheumatoid arthritis [49]. Combined with the observation of increased albumin content in CSF, these results, suggest disruption of the blood brain barrier. These findings all are consistent with an environmental response that is initiated at the hyperplasia/microtumor stage, similar to what has been reported to occur early in systemic cancers such as those involving the breast and prostate [3].

The challenge at this juncture is thus the identification of which components of this host reaction are synergistic and antagonistic relative to favoring tumor growth. Further analysis of these host-tumor environmental interactions should therefore hopefully lead to further insights into these relationships so as to suggest novel therapies that could either prevent tumor formation or treat established tumors.

## Materials and Methods

All experiments performed in this study were approved by the Stanford IUCAC (Protocol #11936) and are in accordance with guidelines for animal safety and welfare.

### ENU Administration

Ethylnitrosourea (ENU) 50 mg/kg was administered to timed pregnant Sprague-Dawley rats (Taconic Farms) at gestational day 18 (E18). Subjects were placed in a restrainer and injected i.p. with 50 mg/kg ENU (Sigma) with a 26-guage needle over a several minute period, as described previously [10]. During each experiment, control pregnant rats received saline vehicle under identical conditions. Pups were born on the appropriate day (usually E23) and appeared normal and healthy. They were weaned at the appropriate time, after which they were housed two per cage.

### Collection of Rat CSF

To obtain CSF for analysis, rats aged between 30 and 90 days were anesthetized with ketamine and xylazine and placed in a stereotactic headholder. Using methodology similar to that described by Pegg *et al*, [50], the dura covering the cisterna magna was exposed by reflecting the posterior neck muscles until the dural surface was detected. The CSF space was then penetrated with a glass pipette using a stereotactic cannula holder, and fluid collected by gentle suction. Routinely 150–250 µl were collected over 30–60 minutes depending on the age of the animal. CSF samples were then immediately centrifuged at 4,000×g for two minutes to remove cells and stored at −80°C until analyzed. Once CSF was obtained, the pipette was removed, and the rat deeply anesthetized and perfused with 4% paraformaldehyde.

### Histological Analysis

After perfusion, brains were removed and prepared for histological analysis by immersion in gradually increasing concentrations of sucrose. Frozen sections were cut spanning an area from the anterior *callosum* to the posterior midbrain and every twelfth section stained with H&E and immunostained with anti-nestin antibody (BD Biosciences Pharmingen) to assess for the presence of hypercellular microtumors and tumor precursors (i.e., nests), respectively, as described previously [10], [12]. The number of single-cell nests, multi-cell nests and microtumors were enumerated on brain sections spaced every 1.2 mm.

### Mass Spectra of Unfractionated Rat CSF

Samples of CSF (6 µl) were denatured with 9 µl 9 M urea/2% CHAPS/0.05 M Tris pH 9 and incubated on ice for 30 minutes. An aliquot of denatured CSF (10 µl) was then added to 90 µl 0.1 M acetate buffer, pH 4, and incubated on a negatively charged weak cation exchange ProteinChip array (CM10, Bio-Rad, Hercules, CA). After binding for 30 minutes, the CM10 arrays were washed three times with binding buffer, and then twice briefly with water. After air-drying, a small amount (1 µL) of the energy absorbing molecule sinapinic acid (5 mg/ml in 50% acetonitrile/0.5% trifluoracetic acid; Bio-Rad) was added to the CM10 arrays and air-dried. After a second application of sinapinic acid and air-drying, the CM10 arrays were placed in a Bio-Rad PCS4000 SELDI mass spectrometer. Spectra were acquired using low-, and medium-, and high-laser energies, which were chosen to optimize for analysis of small (m/z 2,000–10,000), medium (m/z 5,000–30,000) and large (m/z 20,000–200,000) proteins, respectively. The data from all spectra were stored in a MySQL database, for analysis by a variety of techniques (see below).

### Fractionation of Rat CSF Prior to Analysis by SELDI Mass Spectrometry

Some analyses of the P90 rat CSF samples were performed on CSF that was fractionated by strong anion exchange chromatography prior to SELDI analysis. CSF (50 µL) was mixed with 75 µL 9 M urea/2% CHAPS/0.05 M Tris pH 9 and incubated on ice for 30 minutes. After addition of 125 µL 1 M urea/0.2% CHAPS/0.05 M Tris pH 9, the denatured CSF was applied to 50 µL packed HyperD F Q anion exchange beads in a 96 well Silent Screen filtration plate. After agitation for 60 minutes at room temperature, the pass-through fraction was obtained by vacuum filtration and combined with a subsequent wash with 100 µL 0.05 M Tris pH 9. The beads were then successively extracted with two 100 µL washes of buffers with decreasing pH (pH 7, pH 5, pH 4) and a final organic extraction with 33% isopropyl alcohol/17% ACN/0.1% TFA.

For SELDI analysis, aliquots (50 µL) of the pH 9 F×1 fraction were mixed with 50 µL 2 M acetic acid and incubated on CM10 ProteinChip Arrays. After binding of the samples for 30 minutes, the CM10 arrays were washed with 0.1 M acetate buffer pH 4.0 and prepared for mass spectrometry as above.

### Feature Extraction and Analysis of Mass Spectra

The mass spectra were processed using the ProteinChip Data Manager software version 3.5 from the vendor (Bio-Rad). The spectra were de-noised, filtered and baseline subtracted. The processed spectra were then grouped by the age of the rat for normalization of signal intensities. The age-grouped spectra were then recombined for feature extraction (peak finding). The tables of peaks at each age are then used for statistical and biomarker discovery purposes (see below).

### Statistical Analyses

Mann Whitney *U* tests were used to rank differences between peak intensities in CSF from ENU-exposed and control brains. Given the large number of peaks being tested for differential expression, multiple hypotheses testing methods were utilized during the analysis of mass spectra peak intensities. Methods based on false discovery rates (FDR) were used in this analysis (http://translationalmedicine.stanford.edu/Mass-Conductor/FDR.html) [51]. Briefly, global false discoveries and false discovery rate were obtained by randomly permuting the rat identities 100 times, determining the number of discovered peaks each time, and obtaining average number of discovered peaks at each *p* value. Local false discoveries and rates were determined by permuting the rat identities for each peak 100 times.

Chi-square analysis was used for evaluation of nests and early tumors. Student’s *t*-tests were used to evaluate differences in Western blot and immuno-slot blot assays.

Finally, a LASSO analysis was performed to determine the peaks that best discriminated between the ENU group and the control group. This LASSO was computed in Matlab on a standard laptop computer.

### Identification of Differentially Expressed Proteins

See **Supporting Information S1**.

### Western Blotting

CSF samples (4 µL) were subjected to SDS-PAGE under reducing conditions on Bio-Rad TGX 4–20% gradient gels. Proteins were transferred to PVDF membranes in a Thermo semi-dry electro-transfer apparatus. Membranes were blocked with 6% nonfat mild powder in tris-buffered saline pH 7.4 (TBS) (BLOTTO), and incubated with anti-albumin antibody. After washing with TBS supplemented with 1% Tween 20 (TBS/Tween), membranes were incubated with horse radish peroxidase-conjugated goat anti-rabbit IgG (Santa Cruz Biotechnology, Santa Cruz, CA). After washing with TBS/Tween, the blots were visualized after exposure to chemiluminescent substrate SuperSignal Pico (Thermo/Pierce). The band intensities on X-ray film were analyzed using Quantity One software (Bio-Rad).

### Immuno-slot Blotting

PVDF membranes were placed in a Hoefer 48-well slot blot vacuum manifold. The membranes were first treated with 0.5 ml water:methanol (1∶1), and then 0.5 ml TBS twice. Aliquots of CSF (3 µL) were then added to 0.25 ml TBS in each well, and the vacuum applied. The wells were then washed two times with 0.5 ml TBS, after which the membranes were removed and allowed to dry. The membranes were blocked with BLOTTO as above, and then incubated with anti-PGD2S (Santa Cruz Biotechnology), secondary antibody, and chemiluminescent substrate as above. The chemiluminescence intensities were quantified using ImageLab software on a Bio-Rad ChemiDoc XRS+imager.

## Supporting Information

Figure S1 **The effect of increasing blood contamination of CSF on the intensity of the SELDI peaks.** The intensity of the m/z 15216 globin is plotted on the left ordinate axis as a function of the ratio of blood added to CSF. The intensities of three additional peaks are plotted on the right ordinate axis for the same samples of blood-doped CSF samples: the m/z 66610 albumin peak, the m/z 13913 glutathionylated-transthyretin peak (TTY-glut), and the m/z 3493 α1-macroglobulin fragment.
(PDF)

Figure S2 **Purification and Identification of PGD2S.** A) Mean±SEM SELDI intensities of m/z 22893 peaks in Control (Ctl, n = 23) and ENU-exposed (ENU, n = 22) rat CSF; B) mass spectrum of pH 4 fraction showing partial purification of the m/z 22893 peak; C) mass spectrum of proteins extracted from candidate band for the m/z 22893 peak. The change in peak m/z 22975 probably reflects an acrylamide modification of the protein during SDS-PAGE; D) mass spectrum of in-gel reduced/alkylated and trypsinized band from (C).
(PDF)

Figure S3 **Purification and Identification of α1-macroglobulin fragment.** A) Mean±SEM SELDI intensitites of m/z 3493 biomarker in Control (Ctl, n = 23) and ENU-exposed (ENU, n = 22) rat CSF; B) mass spectrum of CSF showing the m/z 3493 peak; C) Purification scheme; D) mass spectrum of purified m/z 3493 peak on NP20 ProteinChip arrays; E, F) MS/MS sequence identification following reduction/alkylation and trypsinization of sample from (D), showing the sequence of the 2628.3 and 884.4 ions, respectively.
(PDF)

Figure S4 **Transthyretin and post-translationally modified transthyretin biomarkers.** A) Maldi mass spectrum following in-gel Arg C-digestion of candidate SDS gel band, without reduction and alkylation; B) MALDI mass spectrum of same band from (A), except that the proteins in the gel band were first reduced and alkylated before Arg C digestion.
(PDF)

Supporting Information S1 **Identification of differentially expressed proteins.**
(DOCX)
