# Supplementary figures and images for: Alterations in Cerebrospinal Fluid Proteins in a Presymptomatic Primary Glioma Model
Source: PLoS One. 2012 Nov 19;7(11):e49724. doi: 10.1371/journal.pone.0049724 (PMC3501526; doi:10.1371/journal.pone.0049724)

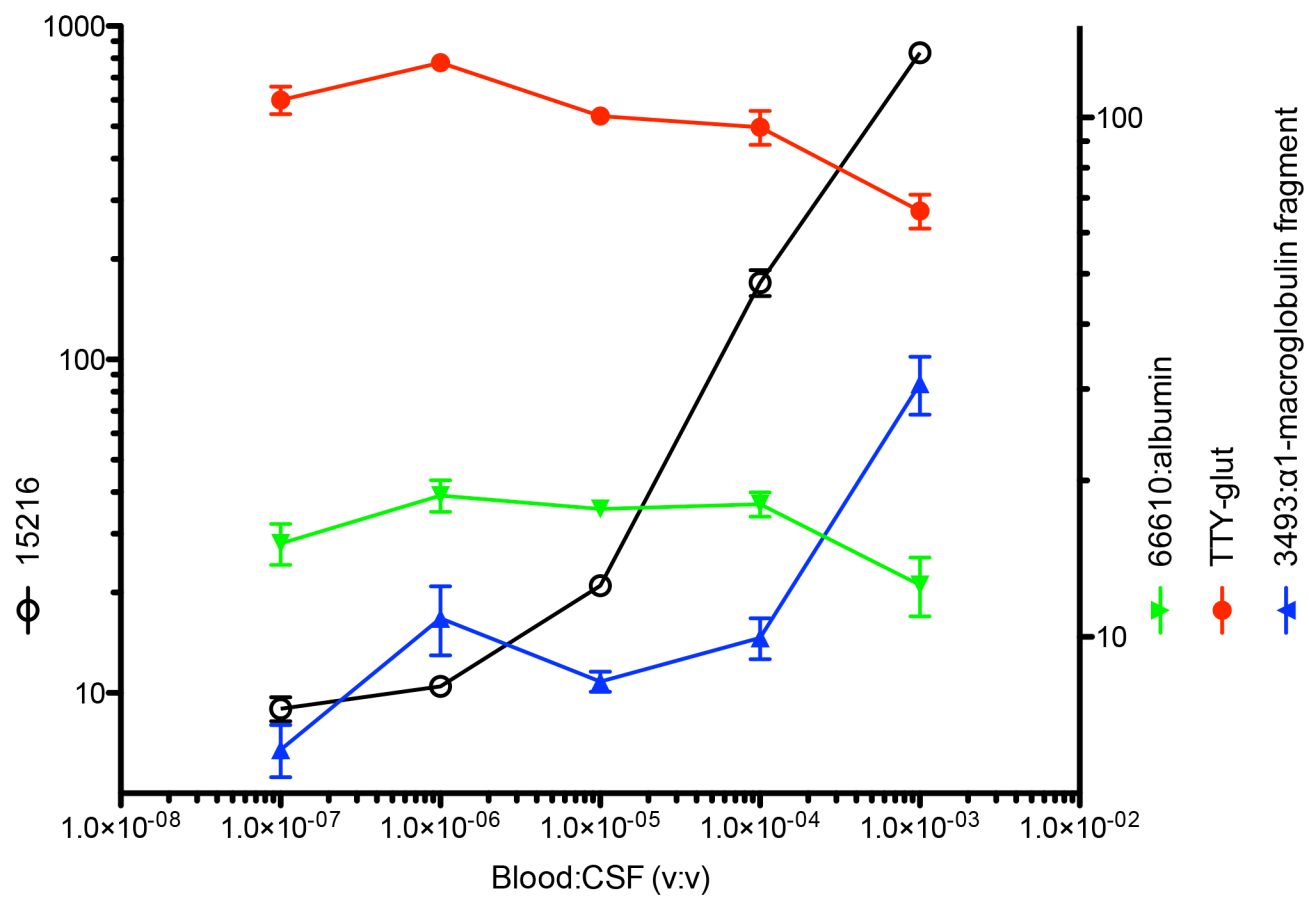

Supplement: Figure S1 — The effect of increasing blood contamination of CSF on the intensity of the SELDI peaks. The intensity of the m/z 15216 globin is plotted on the left ordinate axis as a function of the ratio of blood added to CSF. The intensities of three additional peaks are plotted on the right ordinate axis for the same samples of blood-doped CSF samples: the m/z 66610 albumin peak, the m/z 13913 glutathionylated-transthyretin peak (TTY-glut), and the m/z 3493 α1-macroglobulin fragment. (PDF) [file pone.0049724.s001.pdf]

**A****m/z 22893**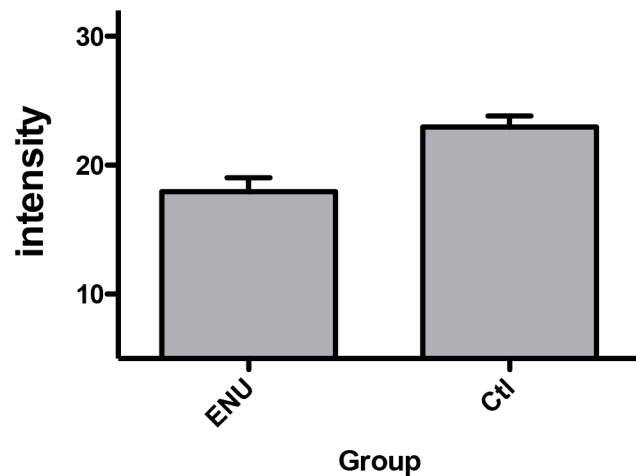**B****m/z 22893**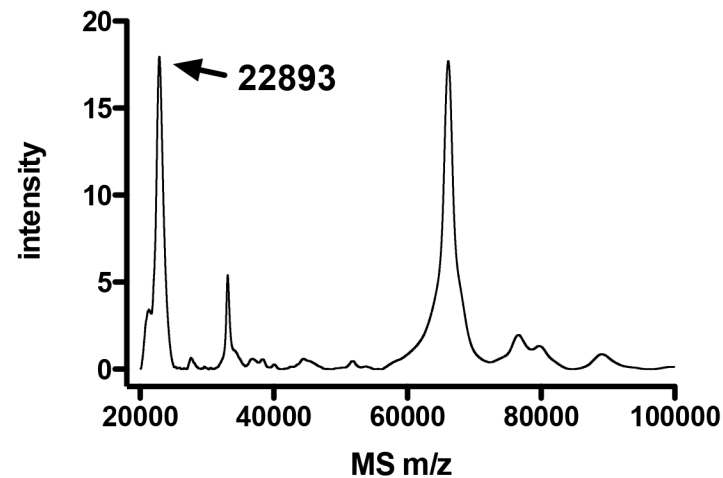**C****MS of purified  
22.9 kDa biomarker band**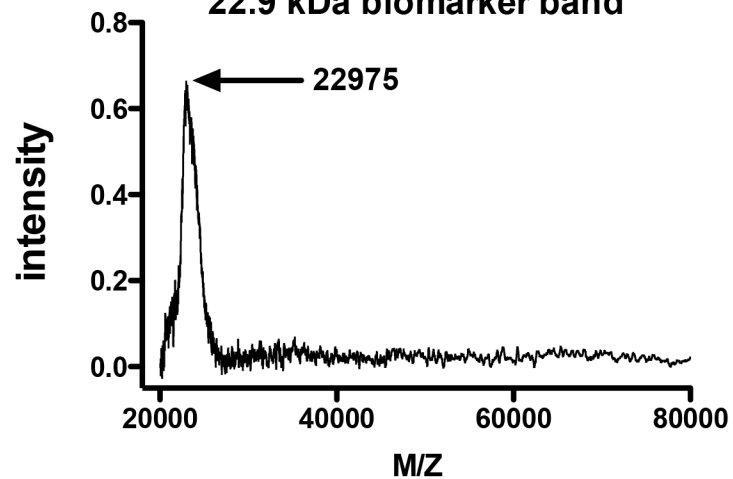**D****MS of trypsinized  
22.9 kDa biomarker band**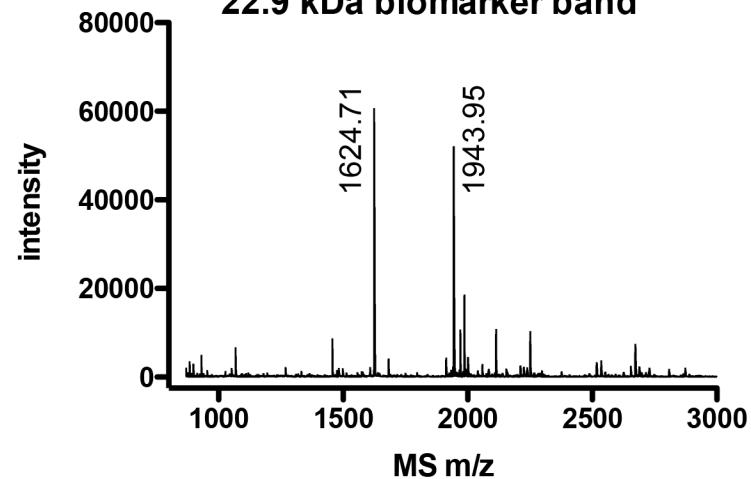

Supplement: Figure S2 — Purification and Identification of PGD2S. A) Mean±SEM SELDI intensities of m/z 22893 peaks in Control (Ctl, n = 23) and ENU-exposed (ENU, n = 22) rat CSF; B) mass spectrum of pH 4 fraction showing partial purification of the m/z 22893 peak; C) mass spectrum of proteins extracted from candidate band for the m/z 22893 peak. The change in peak m/z 22975 probably reflects an acrylamide modification of the protein during SDS-PAGE; D) mass spectrum of in-gel reduced/alkylated and trypsinized band from (C). (PDF) [file pone.0049724.s002.pdf]

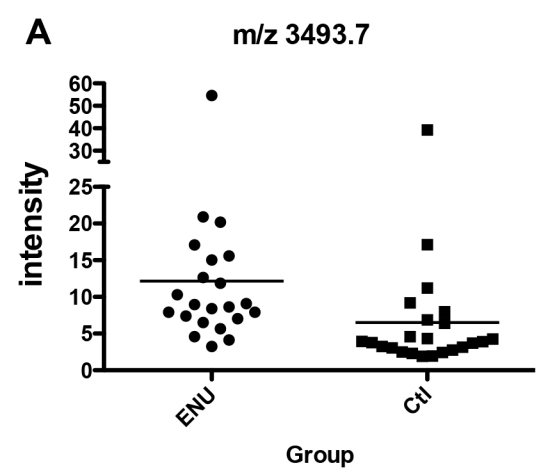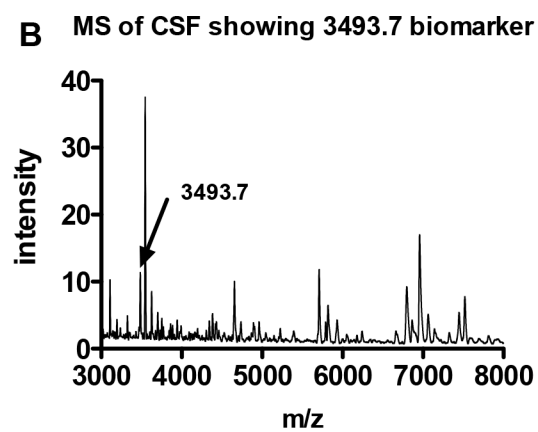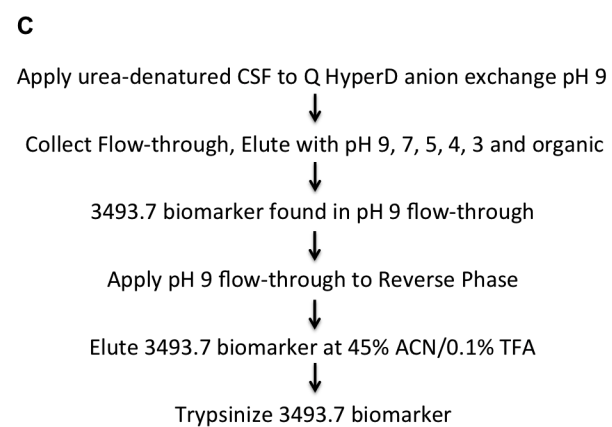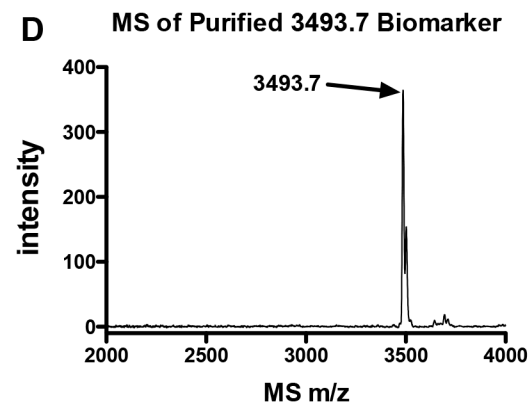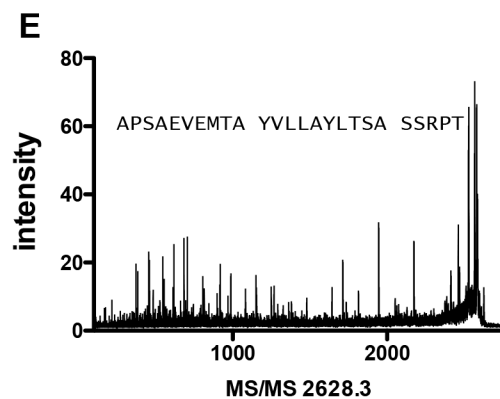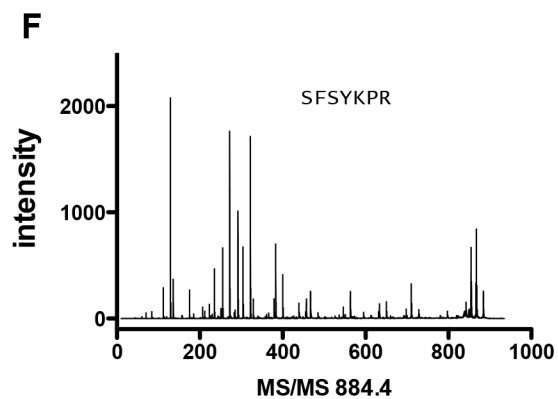

Supplement: Figure S3 — Purification and Identification of α1-macroglobulin fragment. A) Mean±SEM SELDI intensitites of m/z 3493 biomarker in Control (Ctl, n = 23) and ENU-exposed (ENU, n = 22) rat CSF; B) mass spectrum of CSF showing the m/z 3493 peak; C) Purification scheme; D) mass spectrum of purified m/z 3493 peak on NP20 ProteinChip arrays; E, F) MS/MS sequence identification following reduction/alkylation and trypsinization of sample from (D), showing the sequence of the 2628.3 and 884.4 ions, respectively. (PDF) [file pone.0049724.s003.pdf]
