# Supplementary material for: Alterations in Cerebrospinal Fluid Proteins in a Presymptomatic Primary Glioma Model
Source: PLoS One. 2012 Nov 19;7(11):e49724. doi: 10.1371/journal.pone.0049724 (PMC3501526; doi:10.1371/journal.pone.0049724)
Supplement: Figure S4 — Transthyretin and post-translationally modified transthyretin biomarkers. A) Maldi mass spectrum following in-gel Arg C-digestion of candidate SDS gel band, without reduction and alkylation; B) MALDI mass spectrum of same band from (A), except that the proteins in the gel band were first reduced and alkylated before Arg C digestion. (PDF) [file pone.0049724.s004.pdf]

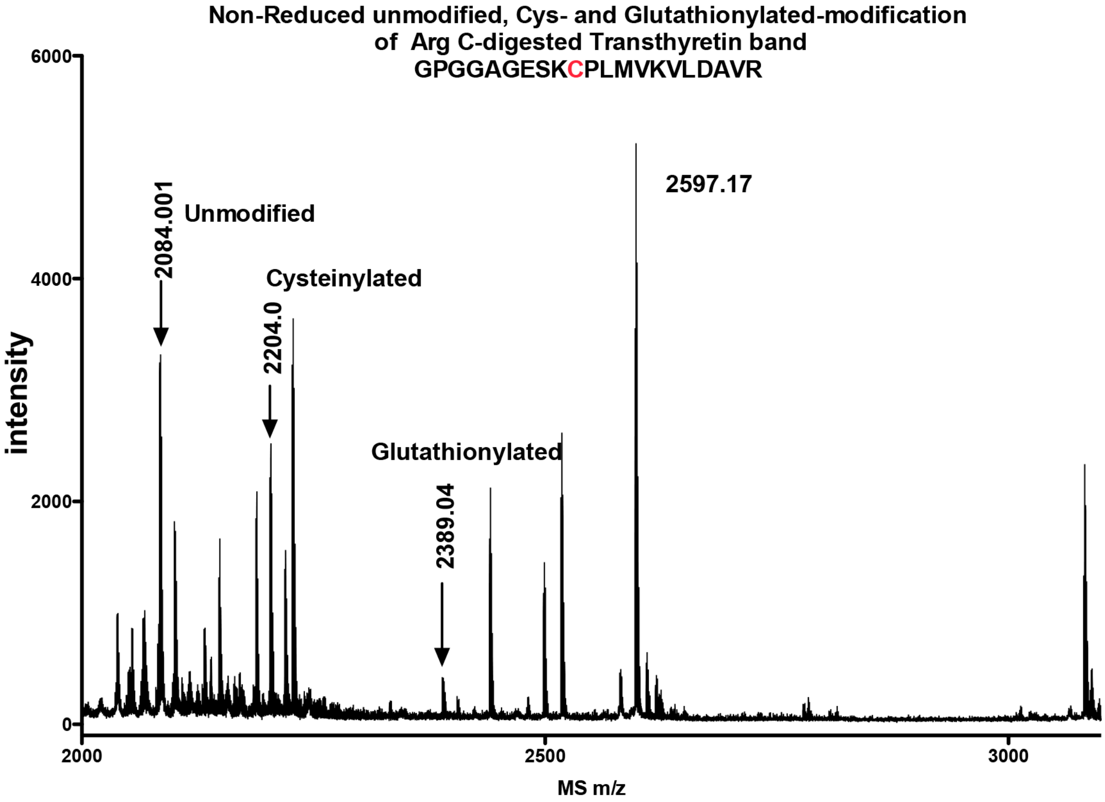
Non-Reduced unmodified, Cys- and Glutathionylated-modification
of Arg C-digested Transthyretin band
GPGGAGESKCPLMVKVLDAVR
6000
4000
2000
0
intensity
2084.001
Unmodified
Cysteinylated
2204.0
Glutathionylated
2389.04
2597.17
2000
2500
3000
MS m/z

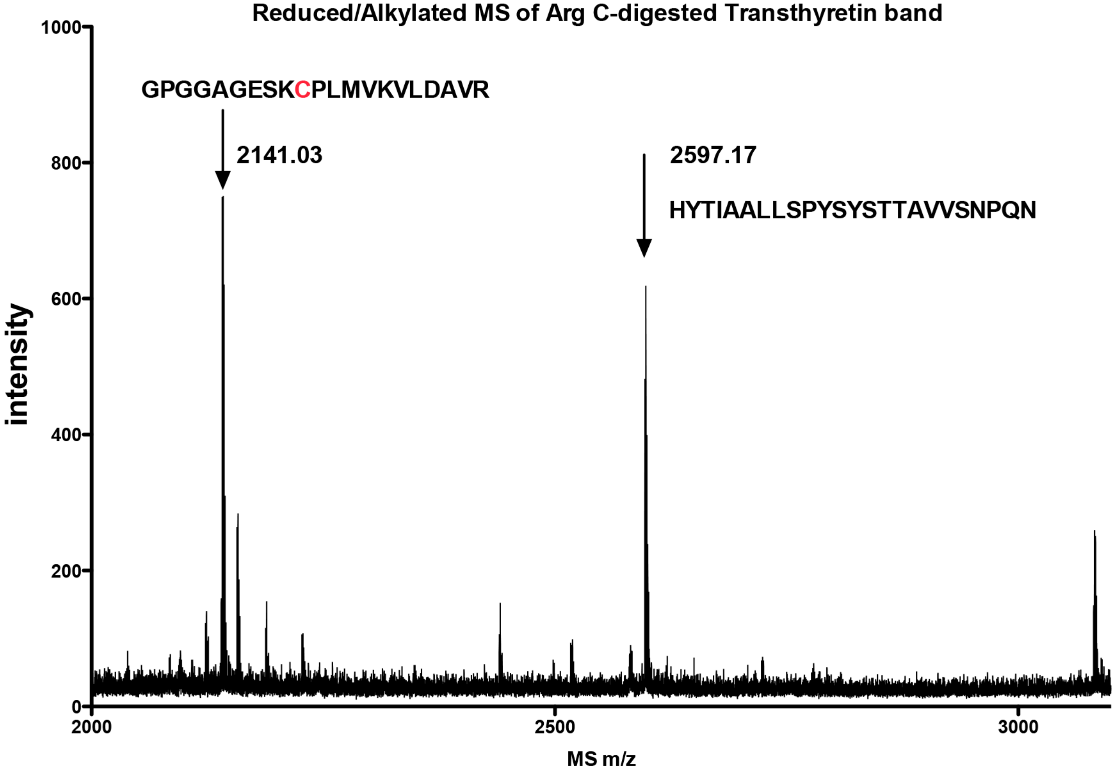
Reduced/Alkylated MS of Arg C-digested Transthyretin band
1000
800
600
400
200
0
intensity
GPGGAGESKCPLMVKVLDAVR
2141.03
2597.17
HYTIAALLSPYSYSTTAVVSNPQN
2000
2500
3000
MS m/z
