# Supplementary material for: Alterations in Cerebrospinal Fluid Proteins in a Presymptomatic Primary Glioma Model
Source: PLoS One. 2012 Nov 19;7(11):e49724. doi: 10.1371/journal.pone.0049724 (PMC3501526; doi:10.1371/journal.pone.0049724)
Supplement: Supporting Information S1 — Identification of differentially expressed proteins. (DOCX) [file pone.0049724.s005.docx]

**SUPPORTING INFORMATION S1**

**Assessment of the Impact of Blood Contamination on CSF Protein Analysis.**

All samples were collected from the cisterna magna to minimize differences in protein concentrations that occur as a function of where CSF is collected. Furthermore, since contamination with even small amounts of blood can cause significant changes in protein concentration because of a 200- to 400-fold higher in protein concentration than CSF as well as the presence of proteases that can produce profound effects on protein analysis ([You, Gelfanova et al. 2005](#_ENREF_34)), we first determined the threshold at which blood contamination starts to affect protein analysis.

Since no RBC’s are normally present in CSF, there is little or no globin present in unadulterated CSF. Since globin peaks are easily recognized on SELDI/TOF, it is possible to monitor blood contamination of CSF. We therefore assessed the effect of doping CSF by adding increasing amounts of blood to CSF, and measuring both globin SELDI peak intensity, and its effect on other peak intensities. EDTA-anticoagulated rat blood (1 mL) was added to 100 ml water to lyse the blood. An aliquot of the hemolyzed blood was then diluted 1:10 with rat CSF, and then serially diluted further with rat CSF to obtain mixtures that represented blood:CSF ratios of 1 x 10 ^-3^ to 1 x 10 ^-7^ . The mixtures were then frozen, thawed, and denatured with urea (4 μL CSF plus 6 μL 9 M urea/2% CHAPS, 0.05 M Tris at pH 9.0). After 30 minutes on ice, the denatured CSF was diluted with 9 volumes of 0.1 M sodium acetate buffer pH 4.0 and bound to CM10 ProteinChip arrays. After washing in acetate buffer, water and application of matrix, mass spectra were collected. This revealed that as little as 0.01% v/v contamination (0.01 μL blood/ml CSF) was enough to measure an increase in globin peak intensities (m/z 15216) (**Figure S1**). Also plotted in **Figure S1** are the peak intensities for three of the biomarkers of interest from the ENU experiments. There was no significant impact of the amount of blood in the CSF until the globin peak intensities increased to >100 μA (at a blood:CSF ratio > 1 x 10 ^-4^ ). For all the CSF samples that were used in the ENU exposure experiments, the globin peak intensities were <20 μA. Thus the possible contamination of these CSF samples was at a level of blood:CSF of 1 x 10 ^-5^ .

**Identification of Candidate Biomarker Proteins.**

Identification of the m/z 22893 peak as PGD2S: CSF (0.2 ml) was mixed with 0.3 ml 9 M urea/2% CHAPS, 0.05 M Tris at pH 9.0 and applied to Q HyperD F strong anion exchange beads (Pall) in a microcentrifuge tube spin column. After mixing for 30 minutes, the flow through was collected, and the beads were successively extracted with buffers at pH 9.0, 7.0, 5.0, 4.0, 3.0 and a final organic wash (33% isopropanol/16.7% acentonitrile (ACN)/0.1% trifluoracetic acid (TFA). The 22.9 kDa peak was primarily found in the pH 4.0 fraction (**Figure S2 B**). This fraction was adjusted to 5% ACN/0.25% (TFA) and applied to polystyrene-divinylbenzene reverse phase beads (PLRP-S; Polymer Laboratories). After mixing for 30 minutes, the beads were successively extracted with increasing concentrations of ACN. An aliquot (1.0 μl) of each extraction was dried onto NP20 ProteinChip arrays. After drying, application of sinapinic acid, and analysis by mass spectrometry, the 22.9 peak was found predominantly in the 40% ACN fraction, and after drying, was solubilized in SDS sample buffer under non-reducing conditions and subjected to SDS-PAGE.

After staining with colloidal Coomassie blue (Invitrogen) and destaining, the candidate bands were excised. A portion of the band was extracted with 50% formic acid, 25% ACN, 15% isopropyl alcohol, 10% H_2_O (FAPH) and the extract applied to an NP20 ProteinChip array for verification of the presence of the protein (**Figure S2 C**). The remaining portions of the band containing the protein were reduced and alkylated, and then subjected to in-gel trypsin digestion. The digest was mixed with CHCA matrix (α-Cyano-4-hydroxycinnamic acid) and MALDI MS spectra acquired on an Applied Biosystems 4700 mass spectrometer. Two major ions were present (**Figure S2 D**): 1624.71 and 1943.95. MS/MS analysis of the 1943.92 ion yielded the sequence DQGLTEEDIVFLPQPDK indicating that the 22.9 kDa biomarker is prostaglandin D2 synthase (P22057). In rat CSF, this protein has two N-glycolsylation sites. Partial sequence information was obtained on the 1624.71 ion that indicated it is likely to be the tryptic fragment containing one of the N-glycosylation sites.

**Identification of the m/z 3493 peak:** The differences between the ENU and Ctl peak intensities for the m/z 3493 peak are shown in **Figure S3 A**. The m/z 3493 biomarker was difficult to purify. A combination of anion exchange chromatography followed by reverse phase chromatography proved fruitful for obtaining enough sample for identification.

Pooled rat CSF (1600 μL) was denatured with 2400 μL 9 M urea, 0.05 M Tris pH 9 (without CHAPS detergent) and incubated on ice for 30 minutes, mixed with an equal volume of 1 M urea/0.05 M Tris pH 9 and applied to Q HyperD strong anion exchange beads at pH 9 in a polypropylene test tube. After tumbling for 60 minutes, the tube was centrifuged and the supernatant was collected. The beads were then washed with 0.5 ml of a series of buffers at pH 9, 7, 5, 4, 3, and an organic extraction as above. An aliquot of each of the fractions (25 μl of unbound fraction, 5 μl of the other fractions) was incubated at pH 4 in 0.1 acetate buffer (final concentration) on CM10 ProteinChip arrays for 30 minutes. After washing, drying, and application of matrix, mass spectra were acquired on the SELDI mass spectrometer. The m/z 3493 peak was found only in the unbound fraction (pH 9) (**Figure S3 B**). The detergent CHAPS could not be used in the original urea denaturation step because it interfered with MALDI mass spectrometry, the method used to monitor the subsequent purification steps.

Numerous attempts to further purify the m/z 3493 biomarker using either cation exchange or C18 reverse phase chromatography failed. This peptide would never elute from weak cation exchange CM media even using pH 10 buffers plus 2 M NaCl, nor from a range of C8 and C18 reverse phase media using up to 95% ACN/0.1% TFA. Instead, the unbound fraction (8 ml) was adjusted to 5% ACN/0.25% TFA and applied to a Macrotrap reverse phase trap column (Michrom) containing 50 μL PLRP-S reverse phase beads, using a syringe pump. The column was then washed with 250 μl 5% ACN/0.1% TFA, followed by 100 μl each of 10%, 20%, 25%, 30%, 35%, 40%, 45%, 50%, and 75% ACN/0.1% TFA, collecting each fraction. One μL of each fraction was then dried onto NP20 ProteinChip arrays. Mass spectra of the fractions showed that the m/z 3493 peak was predominantly found in the 45% ACN fraction (**Figure S3 D**).

The volume of the 45% ACN fraction was reduced in a centrifugal vacuum concentrator, and mixed with CHCA matrix. On an Applied Biosystems 5800 mass spectrometer, the prominent m/z 3493 peak was subjected to direct MS/MS sequencing. An incomplete sequence of a peptide from rat α-1-macroglobulin (Q63041) was obtained by direct sequencing of the m/z 3493 peak (SFSYKPRAPSAEVEMTAYVLLAY). This identification was verified following a second purification of the m/z 3493 biomarker, with and without trypsin digestion. Two peptides were obtained following trypsin digestion: APSAEVEMTAYVLLAYLTSASSRPT (m/z 2628.3)(**Figure S3 E**) and SFSYKPR (m/z 884.4)(**Figure S3 F**). The sequence SFSYKPRAPSAEVEMTAYVLLAYLTSASSRPT thus verified the biomarker to be a novel fragment of rat α-1-macroglobulin. The peptide consists of amino acids 1212-1243 of the unprocessed rat α-1-macroglobulin (1500 amino acids). A 45 kDa subunit of alpha-1-macroglobulin has been reported, from amino acids 1245-1500. Amino acid 1244 in the database is arginine. We did not find evidence of a peptide with an m/z 3650 in either the discovery spectra, or during the purification of the m/z 3493 biomarker; m/z 3650 would be the theoretical m/z of the m/z 3493 peptide with an additional C-terminal arginine.

**Purification and Identification of Transthyretin:** Several peaks with m/z between 13,600 and 14,000 were significantly different by Mann Whitney *U*-test, with low local FDR. To identify these biomarkers, CSF was subjected to anion exchange fractionation. CSF (0.2 ml) was mixed with 0.3 ml 9 M urea/2% CHAPS, 0.05 M Tris at pH 9.0 and applied to Q HyperD F strong anion exchange beads in a small centrifuge tube spin column. After mixing for 30 minutes, the flow through was collected, and the beads were successively washed with buffers at pH 9.0, 7.0, 5.0, 4.0, 3.0 and a final organic wash as above. An aliquot of each fraction was applied to CM10 ProteinChip arrays at pH 4.0 using the same procedures used for biomarker discovery. The biomarker peaks were predominantly found in the pH 4 fraction (Fx4). After reverse phase chromatography using PLRP-S beads of the pH 4 fraction, the biomarker peaks were predominantly found in the 40% ACN fraction. This fraction was then dried, solubilized in SDS sample buffer and subjected to SDS-PAGE under non-reducing conditions. The range of peaks was extracted from the candidate band using FAPH, and the remainder of the band was subjected to in-gel trypsin digestion after reduction and alkylation of the band fragments. After MALDI MS of the trypsin digest and subsequent MS/MS analysis of predominant ions, peptides from transthyretin (P02767) were identified. The peptide HYTIAALLSPYSYSTTAVVSNPQN was identified from the 2597.16 ion (not shown). Transthyretin is known to exist in plasma in multiple post-translationally-modified forms. The tenth amino acid of the processed form of rat transthyretin is cysteine. This cysteine residue can exist in unmodified, cysteinylated, glutathionylated, and other oxidized forms. To test whether our range of biomarker peaks correlated with these oxidized forms of transthyretin, we purified more of the m/z 13,600-14,000 biomarkers from rat CSF. After SDS-PAGE and excision of the biomarker band, the band was split into 3 pieces. One piece was extracted with FAPH to verify the presence of the biomarker peaks. One piece was digested directly with Arg C endopeptidase, while the third piece was first reduced and alkylated before digestion with Arg C. While trypsin digests at two sites very near the Cys residue yielding only a six amino acid peptide, Arg C is predicted to generate a 21 amino acid peptide that contains the Cys. Both the non-reduced and reduced/alkylated ArgC digests were then subjected to MS and MS/MS analysis.

In the MS spectrum of the non-reduced sample, the m/z 2084.0, 2204.0, and 2389.0 peaks are consistent with the unmodified, cysteinylated, and glutathionylated forms of the peptide GPGGAGESKCPLMVKVLDAVR from Arg C-digestion of rat transthyretin, respectively (**Figure S4 A**). After reduction and alkylation of the band, the m/z 2141.0 peak was sequenced by MS/MS and identified as the expected carboxamidomethyl modification of the cysteine-containing peptide. In this reduced and alkylated sample, the peaks corresponding to the unmodified, cysteinylated and glutathionylated peptide are no longer present (**Figure 4S B**).

Figure S1. The effect of increasing blood contamination of CSF on the intensity of the SELDI peaks. The intensity of the m/z 15216 globin is plotted on the left ordinate axis as a function of the ratio of blood added to CSF. The intensity of three additional peaks on plotted on the right ordinate axis for the same samples of blood-doped CSF samples: the m/z 66610 albumin peak, the m/z 13913 glutathionylated-transthyretin peak (TTY-glut), and the m/z 3493 α1-macroglobulin fragment.

**Figure S2.** Purification and Identification of PGD2S. A) Mean ± SEM SELDI intensities of m/z 22893 peaks in Control (Ctl, n = 23) and ENU-exposed (ENU, n = 22) rat CSF; B) mass spectrum of pH 4 fraction showing partial purification of the m/z 22893 peak; C) mass spectrum of proteins extracted from candidate band for the 22893 peak. The peak m/z of 22975 probably reflects an acrylamide modification of the protein during SDS-PAGE; D) mass spectrum of in-gel reduced/alkylated and trypsinized band from (C).

**Figure S3.** Purification and Identification of α1-macroglobulin fragment. A) Mean ± SEM SELDI intensitites of m/z 3493 biomarker in Control (Ctl, n = 23) and ENU-exposed (ENU, n = 22) rat CSF; B) mass spectrum of CSF showing the 3493 peak; C) Purification scheme; D) mass spectrum of purified m/z 3493 peak on NP20 ProteinChip arrays; E, F) MS/MS sequence identification following reduction/alkylation and trypsinization of sample from D, showing the sequence of the 2628.3 and 884.4 ions, respectively.

**Figure S4.** Transthyretin and post-translationally modified transthyretin biomarkers. A) Maldi mass spectrum following in-gel Arg C-digestion of candidate SDS gel band, without reduction and alkylation; B) MALDI mass spectrum of same band from (A), except that the proteins in the gel band were first reduced and alkylated before Arg C digestion.
